# Supplementary material for: A Meta-Analysis to Estimate Prevalence of Resistance to Tetracyclines and Third Generation Cephalosporins in Enterobacteriaceae Isolated from Food Crops
Source: Antibiotics (Basel). 2022 Oct 17;11(10):1424. doi: 10.3390/antibiotics11101424 (PMC9598472; doi:10.3390/antibiotics11101424)
Supplement: Supplementary file 1 [file antibiotics-11-01424-s001.zip › antibiotics-1916167-supplementary.pdf]

Supplementary Materials

Study	Events	Total	Weight	IV, Random, 95% CI
Reddy et al 2016	2	139154	2.5%	0.0000 [0.0000; 0.0001]
Van Hoek et al 2015	1	1188	2.5%	0.0008 [0.0000; 0.0047]
Kaesbohrer et al 2019	1	399	2.5%	0.0025 [0.0001; 0.0139]
Haile et al 2021	1	390	2.5%	0.0026 [0.0001; 0.0142]
Thung et al 2020	2	360	2.5%	0.0056 [0.0007; 0.0199]
Song et al 2020	11	1324	2.5%	0.0083 [0.0042; 0.0148]
Colosi et al 2020	1	165	2.4%	0.0061 [0.0002; 0.0333]
Li et al 2020	1	154	2.4%	0.0065 [0.0002; 0.0356]
Hartantyo et al 2020	1	86	2.2%	0.0116 [0.0003; 0.0631]
Rodrigues et al 2022	2	145	2.3%	0.0138 [0.0017; 0.0489]
Kholdi et al 2021	7	400	2.5%	0.0175 [0.0071; 0.0357]
Holvoet et al 2013	5	264	2.4%	0.0189 [0.0062; 0.0436]
Shahin et al 2019	14	650	2.5%	0.0215 [0.0118; 0.0359]
Najwa et al 2015	2	96	2.3%	0.0208 [0.0025; 0.0732]
Freitag et al 2018	6	245	2.4%	0.0245 [0.0090; 0.0525]
Abakpa et al 2017	6	238	2.4%	0.0252 [0.0093; 0.0541]
Lopes et al 2021	1	48	2.0%	0.0208 [0.0005; 0.1107]
Gomez-Aldapa et al 2016	6	200	2.4%	0.0300 [0.0111; 0.0642]
Romyasamit et al 2021	10	305	2.4%	0.0328 [0.0158; 0.0595]
Niyomdecha et al 2016	1	40	1.9%	0.0250 [0.0006; 0.1316]
Chotinantakul et al 2021	7	182	2.4%	0.0385 [0.0156; 0.0776]
Priyanka et al 2021	37	830	2.5%	0.0446 [0.0316; 0.0609]
Aabed et al 2021	1	30	1.8%	0.0333 [0.0008; 0.1722]
Li et al 2020	7	154	2.4%	0.0455 [0.0185; 0.0914]
Skockova et al 2014	4	91	2.2%	0.0440 [0.0121; 0.1087]
Kurittu et al 2021	3	60	2.1%	0.0500 [0.0104; 0.1392]
Janalikova et al 2018	6	108	2.3%	0.0556 [0.0207; 0.1170]
Zou et al 2019	1	23	1.7%	0.0435 [0.0011; 0.2195]
Al Kharousi et al 2022	2	39	1.9%	0.0513 [0.0063; 0.1732]
Rasheed et al 2014	2	30	1.8%	0.0667 [0.0082; 0.2207]
Zekar et al 2017	44	491	2.5%	0.0896 [0.0659; 0.1184]
Lopes et al 2021	4	48	2.0%	0.0833 [0.0232; 0.1998]
Pintor-Cora et al 2021	11	117	2.3%	0.0940 [0.0479; 0.1620]
Kim et al 2015	19	189	2.4%	0.1005 [0.0616; 0.1525]
Montero et al 2021	24	236	2.4%	0.1017 [0.0663; 0.1475]
Mwanza et al 2021	6	51	2.0%	0.1176 [0.0444; 0.2387]
Somda et al 2021	10	78	2.2%	0.1282 [0.0632; 0.2232]
Ananchaipattana et al 2013	5	38	1.9%	0.1316 [0.0441; 0.2809]
Chanseyha et al 2018	9	60	2.1%	0.1500 [0.0710; 0.2657]
Richter et al 2020	31	192	2.4%	0.1615 [0.1124; 0.2213]
Sun et al 2021	53	264	2.4%	0.2008 [0.1541; 0.2542]
Campos et al 2013	11	50	2.0%	0.2200 [0.1153; 0.3596]
Kabir et al 2014	3	14	1.4%	0.2143 [0.0466; 0.5080]
Chanseyha et al 2018	19	60	2.1%	0.3167 [0.2026; 0.4496]
Guran et al 2022	4	12	1.3%	0.3333 [0.0992; 0.6511]
**random effects model**		**149298**	**100.0%**	**0.0475 [0.0292; 0.0694]**

Heterogeneity: $Tau^2 = 0.0201$; $Chi^2 = 1619.22$, df = 44 ($P < 0.01$); $I^2 = 97\%$

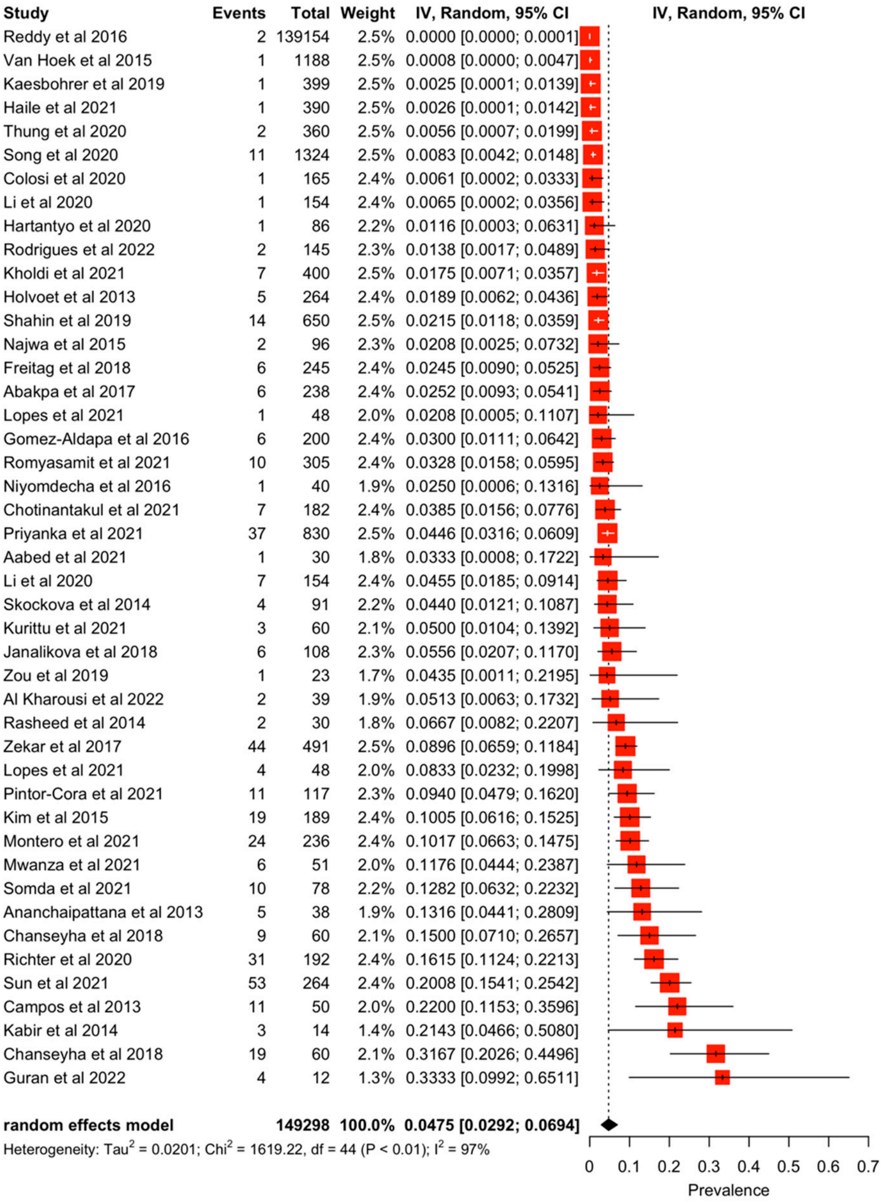

Figure S1: Forest Plot of Citations with Tetracycline and Third-Generation Cephalosporin Resistant Prevalence Estimates. The column titled ‘Events’ are the number of plant-food samples that were reported resistant to tetracycline and/or third-generation cephalosporin antimicrobials. The column titled ‘Total’ are the number of plant-food samples selected for antimicrobial susceptibility testing.

Table S1: Table of Included Citations.

Study	Country	Sample	Stage	Microbe name	Sample weight (g)	Interpretive Criteria	AMR	Resistant Samples	Total Samples	Prevalence (%)
Reddy *et al.* 2016	USA (imported)	green onion, lettuce	Post-harvest	*Salmonella* spp.	NA	Not reported	TET	2	139,154	0.001
van Hoek *et al.* 2016	Netherlands (imported)	celery, carrots, lettuce, endive, radish	Post-harvest	Enterobacteriaceae	60	CLSI (2012)	3GC	1	1,188	0.1
Kaesbohrer *et al.* 2019	Germany	vegetables	Post-harvest	*Escherichia coli*	25	CLSI (2013)	3GC	1	399	0.25
Haile *et al.* 2021	Ethiopia	lettuce	Post-harvest	*Escherichia coli*	25	CLSI (2014)	TET	1	390	0.25
Thung *et al.* 2020	Malaysia	brinjal, cucumber, ladies fingers	Pre-harvest	*Salmonella* spp.	25	CLSI (2012)	TET	2	360	0.56
Song *et al.* 2020	South Korea	leafy vegetables	Post-harvest	*Escherichia coli*	30	CLSI (no year reported)	3GC	11	1,324	0.83
Colosi *et al.* 2020	Romania	cucumbers	Post-harvest	*Escherichia coli*	2	EUCAST	3GC	1	165	0.6
Li et al 2020	China	bok choy, broccoli, bean sprout, celery, lotus root, cabbage, cucumber, clery, garlic sprout, leafy veg, chinese cabbage, black fungus	Post-harvest	*Escherichia coli*	25	EUCAST (2017)	3GC	1	154	0.006

Hartantyo *et al.* 2020	Singapore	bean sprouts, spring onions, chilis, parsley, lettuce, cucumbers, carrots, ginger	Post-harvest	*Klebsiella pneumonia*	10	CLSI (2014)	3GC	1	86	1.2
Rodrigues *et al.* 2022	Denmark, Ireland, Italy, France, Austria	salad leaf samples	Post-harvest	*Klebsiella pneumonia*	NA	EUCAST	TET	2	145	1.37
Kholdi *et al.* 2022	Iran	carrots, lettuce, cucumber, leafy greens	Post-harvest	*Escherichia coli*	25	CLSI (2017)	3GC	7	400	1.75
Holvoet *et al.* 2013	Belgium	lettuce	Pre-harvest	*Escherichia coli*	10	CLSI (2011)	TET	5	264	1.9
Shahin *et al.* 2019	Iran	vegetables	Post-harvest	*Shigella* spp.	10	CLSI (2015)	TET	14	650	2.2
Najwa et al 2015	Malaysia	ulam (asian pennywort, water dropwort, long bean, winged bean)	Post-harvest	*Salmonella* spp.	10	CLSI	TET	2	96	2
Freitag *et al.* 2018	Germany, Netherlands	salad and sprouts	Post-harvest	*Escherichia coli*	25	CLSI (2013, 2015)	TET	6	245	2
Abakpa *et al.* 2017	Nigeria	salad vegetables	Pre-harvest	*Escherichia coli*	10	CLSI (2010)	TET	6	238	2.5
Lopes *et al.* 2021	Brazil	spinach, lettuce, arugula, cabbage	Post-harvest	*Escherichia coli*; *Klebsiella pneumoniae*	4	CLSI (2018, 2020)/EUCAST	TET	1	48	2.1
Gomez-Aldapa *et al.* 2016	Mexico	nopalitos	Post-harvest	*Escherichia coli*	100	CLSI (2009, 2014)	TET	6	200	3

Romyasamit *et al.* 2021	Thailand	Thai yardlong beans, Thai eggplant, winged bean, cashew leaves, basil, cabbage, cucumber, tomato, coriander, lettuce	Post-harvest	*Escherichia coli*; *Klebsiella pneumonia*	25	CLSI (2019)	TET & 3GC	10	305	3.3
Niyomdecha *et al.* 2016	Thailand	lettuce	Post-harvest	*Salmonella* spp.	50	CLSI (2013)	TET	1	40	2.5
Chotinantakul *et al.* 2022	Thailand	spring onion, sweet basil, cha-om, lemongrass, yard long beans, lettuce, holy basil	Post-harvest	*Escherichia coli*	20	CLSI (2019)	3GC	7	182	3.8
Priyanka *et al.* 2021	India	cabbage, coriander, fenugreek, spinach, peppermint	Post-harvest	*Salmonella* spp.	25	CLSI (no year reported	TET & 3GC	37	830	4.5
Aabed *et al.* 2021	Saudi-Arabia	vegetables	Post-harvest	*Escherichia coli*	10	CLSI (no year reported)	3GC	1	30	3.3
Li *et al.* 2020	China	bok choy, broccoli, bean sprout, celery, lotus root, cabbage, cucumber, clery, garlic sprout, leafy veg, chinese	Post-harvest	*Escherichia coli*	25	EUCAST (2017)	TET	7	154	4.5

Skockova *et al.* 2013	Czech Republic (imported)	cabbage, black fungus asparagus, leaf lettuce, caultiflower, cucumber, spring onion, mung sprouts, rucola, leek, radish chinese cabbage	Post-harvest	*Escherichia coli*	NA	CLSI (2006	TET	4	91	4.4
Kurittu *et al.* 2021	Malaysia	Coriander; chili pepper	Post-harvest	*Escherichia coli*; *Klebsiella pneumonia*	25	EUCAST (2017)	3GC	3	60	5
Janalikova *et al.* 2018	Europe	vegetables	Post-harvest	*Escherichia coli*	10	EUCAST (2014)	3GC	6	108	5.5
Zou *et al.* 2019	China	beans, cucumber, peppers, celery, leeks	Pre-harvest	*Escherichia coli*	NA	CLSI (2016)	TET	1	23	4.3
Al-Kharousi *et al.* 2021	Oman	cabbage, lettuce, radish	Post-harvest	*Escherichia coli*	25	CLSI (2015)	TET	2	39	5.1
Rasheed *et al.* 2014	India	carrots, cucumber, tomatoes, spinach, lettuce, beets, radishes	Post-harvest	*Escherichia coli*	25	CLSI (2002)	3GC	2	30	6.67
Zekar *et al.* 2017	Algeria	mixed vegetables	Post-harvest	Enterobacteria ceae	25	CLSI (2013); EUCAST (2013)	3GC	44	491	8.96
Lopes *et al.* 2021	Brazil	spinach, lettuce, arugula, cabbage	Post-harvest	*Enterobacter cloaca*; *Klebsiella pneumonia*; *Escherichia coli*	4	CLSI (2018, 2020)/EUCAST	3GC	4	48	8.3

Pintor-Cora *et al.* 2021	Spain	carrot, coriander, escarole, lettuce, parsley, tomato		Enterobacteriaceae*	10	EUCAST	3GC	11	117	9.4
Kim *et al.* 2015	South-Korea	sprouts and salad	Post-harvest	*Escherichia coli*; *Klebsiella pneumonia*	25	CLSI (2013)	3GC	19	189	10.1
Montero *et al.* 2021	Ecuador	vegetables and fruit	Pre-harvest	*Escherichia coli*	10	CLSI (2019)	TET	24	236	10.2
Mwanza *et al.* 2021	Tanzania	African spinach	Pre-harvest	*Escherichia coli*	10	CLSI (2013)	TET; 3GC	6	51	11.8
Somda *et al.* 2021	Burkina-Faso	lettuce	Pre-harvest	*Salmonella* spp.	NA	EUCAST (2017)	TET	10	78	12.8
Ananchaipattana *et al.* 2014	Thailand	leafy vegetables	Post-harvest	*Salmonella* spp.	25	CLSI (no year reported)	TET	5	38	13.2
Chanseyha *et al.* 2018	Cambodia	green leaf lettuce	Post-harvest	*Escherichia coli*; *Salmonella* spp.	25	CLSI (2013)	TET	9	60	15
Richter *et al.* 2020	South-Africa	cucumber, green beans, lettuce, spinach, tomatoes	Post-harvest	*Escherichia coli*; *Klebsiella pneumoniae*	50	CLSI (2018)	TET & 3GC	31	192	16.1
Sun *et al.* 2021	China	onion, coriander, lettuce, cucumber, melon, grape, peach	Post-harvest	*Escherichia coli*	25	CLSI (2019)/EUCAST (2019)	TET	53	264	20.1
Campos *et al.* 2013	Portugal	ready-to-eat salads	Post-harvest	*Escherichia coli*	25	CLSI (2011)	TET	11	50	22
Kabir *et al.*2014	Bangladesh	cabbage, carrot, turnip	Post-harvest	*Escherichia coli*	10	CLSI (2011)	3GC	3	14	21.4

Chanseyha *et al.* 2018	Thailand	green leaf lettuce	Post-harvest	*Escherichia coli; Salmonella* spp.	25	CLSI (2013)	TET	19	60	31.2
Guran et al 2021	Cyprus	banana, apple, lettuce, carrot	Post-harvest	*Escherichia coli*	40	EUCAST (2015)	TET	4	12	33.3

*The authors of Pintor-Cora *et al*. 2021 used the final taxonomic definition of Enterobactericeae prior to recent revision to the families within the order Enterobacterales.
